# Natural Antimicrobial Mixtures Disrupt Attachment and Survival of *E. coli* and *C. jejuni* to Non-Organic and Organic Surfaces

**DOI:** 10.3390/foods12203863

**Published:** 2023-10-21

**Authors:** Nicolae Corcionivoschi, Igori Balta, Eugenia Butucel, David McCleery, Ioan Pet, Maria Iamandei, Lavinia Stef, Sorin Morariu

**Affiliations:** 1 Bacteriology Branch, Veterinary Sciences Division, Agri-Food and Biosciences Institute, Belfast BT4 3SD, UK; nicolae.corcionivoschi@afbini.gov.uk (N.C.); eugenia.butucel@afbini.gov.uk (E.B.); david.mccleery@afbini.gov.uk (D.M.); 2 Faculty of Bioengineering of Animal Resources, University of Life Sciences King Mihai I from Timisoara, 300645 Timisoara, Romania; balta.igori@usvt.ro (I.B.); ioanpet@usvt.ro (I.P.); lavi_stef@animalsci-tm.ro (L.S.); 3 Academy of Romanian Scientists, Ilfov Street, No. 3, 050044 Bucharest, Romania; 4 Research Development Institute for Plant Protection, 013813 Bucharest, Romania; 5 Faculty of Veterinary Medicine, University of Life Sciences King Mihai I from Timisoara, 300645 Timisoara, Romania

**Keywords:** *Campylobacter jejuni*, *Escherichia coli*, biofilm formation, foodborne pathogens, natural antimicrobials, food industry

## Abstract

The contact and adherence of bacteria to various surfaces has significant consequences on biofilm formation through changes in bacterial surface structures or gene expression with potential ramifications on plant and animal health. Therefore, this study aimed to investigate the effect of organic acid-based mixtures (Ac) on the ability *Campylobacter jejuni* and *Escherichia coli* to attach and form biofilm on various surfaces, including plastic, chicken carcass skins, straw bedding, and eggshells. Moreover, we aimed to explore the effect of Ac on the expression of *E. coli* (*lux*S, *fim*C, *csg*D) and *C. jejuni* (*lux*S, *fla*A, *fla*B) bacterial genes involved in the attachment and biofilm formation via changes in bacterial surface polysaccharidic structures. Our results show that Ac had a significant effect on the expression of these genes in bacteria either attached to these surfaces or in planktonic cells. Moreover, the significant decrease in bacterial adhesion was coupled with structural changes in bacterial surface polysaccharide profiles, impacting their adhesion and biofilm-forming ability. Essentially, our findings accentuate the potential of natural antimicrobials, such as Ac, in reducing bacterial attachment and biofilm formation across various environments, suggesting promising potential applications in sectors like poultry production and healthcare.

## 1. Introduction

The ability to attach to various surfaces ensures the survival and persistence of bacteria in various industrial environments [1]. These sessile bacteria-fortified communities increase bacterial tolerance to various factors, including chemicals (e.g., metals, aldehydes, phenols, biocides, and antibiotics), and reduce the effectiveness of disinfectants, thereby contributing to bacterial persistence or even to the development of multidrug resistance [2,3,4]. Bacteria can form biofilms on both biotic and abiotic surfaces by using their pili and flagella [5]. Subsequently, they proliferate and produce extracellular polymeric substances (EPS), which help them adhere tightly and form a matrix that, owing to its heterogeneous structure, can protect bacteria in the biofilm against antimicrobial agents [6]. Bacteria such as *Campylobacter jejuni* (*C*. *jejuni*) and *Escherichia coli* (*E*. *coli*) are well known for their ability to adhere to the surfaces of biological or medical devices and produce strong biofilms, which can lead to serious complications and chronic infections. Pathogenic bacteria play a crucial role in infectious diseases and are one of the leading causes of death in humans and animals [7]. *E*. *coli* and *C*. *jejuni* are two common pathogenic bacteria responsible for a range of infections, such as urinary tract infections, gastroenteritis, and foodborne illnesses [8]. The formation of biofilms by these bacteria is a major concern in various fields, including healthcare, the food industry, and environmental sciences, contributing to their increased virulence and resistance to antimicrobials and host immune responses [9,10,11]. 

*E*. *coli* and other pathogens can sense and respond to fluctuating population densities via QS, which is crucial for survival. The *luxS* gene, encoding the AI-2 autoinducer synthesis pathway, is a key player in this complex bacterial growth and communication system [12,13]. *Lux*S catalyzes the conversion of ribosyl homocysteine to homocysteine and 4,5-dihydroxy-2,3-pentanedione, the latter spontaneously forming the autoinducer-2 (AI-2) molecule [2]. AI-2, a universal signaling molecule, facilitates interspecies communication, allowing *E*. *coli* to interact with diverse microbial communities. However, based on recent data, AI-2 is frequently a metabolic by-product rather than a signal [14]. Recent findings highlight the identification of a novel signaling entity, AI-3 [15]. Intriguingly, its synthesis appears to be independent of the *lux*S pathway, suggesting a potential role of AI-3 in facilitating interactions between EHEC and the host’s epinephrine–norepinephrine signaling mechanism [15]. Exposure to biocides (e.g., sub-lethal benzalkonium chloride concentrations) has been observed to enhance AI-2 production in *E*. *coli*, thereby strengthening biofilm development, enhancing exopolysaccharide (EPS) synthesis, and elevating swimming motility. Notably, the expression of *lux*S and of genes associated with biofilm and flagellar functions were upregulated in the presence of the biocide, suggesting its unsuitability as a disinfectant in food production spaces to mitigate *E*. *coli* contamination risks [16]. On the contrary, combinations of essential oils (Eos) (e.g., lavandin) had potent antibacterial effects against *C*. *jejuni* [17]. These lavandin preparations decreased *C*. *jejuni* adhesion to polystyrene surfaces and hindered biofilm formation on glass substrates. Moreover, a marked decrease in *C*. *jejuni* intercellular communication was observed with Lavandin Eos, with the most effective preparations reducing this signaling by an estimated 95%. Similarly, biological compounds such as proanthocyanidins and flavanoids from *Rhodiola rosea* PACs were found to decrease intercellular signaling reduction rates of *Campylobacter* by ≈65% and showed bioluminescence lessening rates by ≈70% [18]. 

Transcriptomic analyses have also recently indicated that *csg*D may have a regulatory role, either directly or indirectly, over metabolic processes, cellular membrane integrity, and enzymatic activity [19]. Notably, *csg*D appears to positively modulate genes associated with bacterial flagellar assembly, cellular adhesion, and stress-response mechanisms. Specifically, *csg*D impacted the transcriptional levels of genes such as *pga*A, *mot*B, *fim*A, *fim*C, *ira*P, *omp*A, *osm*C, *suf*E, and *ela*B, all of which are associated with biofilm formation and stress response. The *fim*C gene is integral to the Type 1 fimbriae assembly mechanism in *E*. *coli*, playing an instrumental role in bacterial adherence and colonization. The *fim*C gene encodes for a periplasmic chaperone, facilitating the proper folding of fimbrial subunits, which are crucial for the construction of the Type 1 fimbriae [20]. Without the functional contribution of *fim*C, the biogenesis and stability of these fimbriae are compromised. With functional Type 1 fimbriae, *E*. *coli* can adhere to and colonize various surfaces, especially uroepithelial cells. This is a pivotal virulence factor in the context of urinary tract infections (UTIs). Also, the *fim*C-mediated fimbrial assembly allows *E*. *coli* to interact intimately with host cells, leading to cellular invasion or toxin-mediated damage and contributing to biofilm formation, offering *E*. *coli* enhanced resistance to environmental stressors and antibiotics [21,22]. 

Central to the pathogenicity of *Campylobacter* spp. is their motility, which plays a fundamental role in colonization and host invasion. At the heart of this motility mechanism is the *fla*A gene, encoding a major flagellin protein that forms the filamentous part of the bacterial flagellum [23]. The gene is often co-located with another flagellin gene, *fla*B, though it is the FlaA protein that is more abundant in the flagellar structure. Recent proteomic studies indicated an elevated expression of flagellin proteins (FlaA, FlaB) in biofilm forms of *C*. *jejuni* compared to their planktonic counterparts [24]. Additionally, mutations in the *C*. *jejuni fla*A gene resulted in a loss of motility, leading to diminished biofilm development on food industry surfaces [24]. Mutants of *Campylobacter* that lack a functional *fla*A gene are often significantly less motile. More crucially, they exhibit a dramatic reduction in their ability to colonize and cause infections in hosts [25]. This reduced virulence showcases the importance of *fla*A in the disease process [25]. The adhesion of *Campylobacter* to host cells, an initial step in infection, is also influenced by *fla*A [23]. Its structure and properties may mediate interactions with host cell receptors, facilitating tighter binding and eventual invasion [26]. Exposure of *C*. *jejuni* to trans-cinnamaldehyde resulted in a marked decrease in the expression of motility-associated genes *fla*A, *fla*B, and *flg*A, with maximum reductions observed at approximately ≈11.7 folds, respectively [24]. Contrarily, the expression of the quorum sensing gene (*lux*S), which plays a pivotal role in intercellular communication during biofilm establishment, experienced a significant increase, approximately ≈6 folds. Furthermore, the authors reported that trans-cinnamaldehyde significantly disrupted the biofilm formation and architecture, which released extracellular polymeric compounds [24]. Recent in vitro studies have highlighted the efficacy of certain Anti-Campylobacter probiotics, including *L*. *salivarius*, *L*. *johnsonii*, *L*. *crispatus*, and *L*. *gasseri*, in suppressing the expression of virulence genes in *C*. *jejuni* [27]. These genes encompass those governing motility (such as *fla*A, *fla*B, and *flh*A), cellular invasion (like *cia*B), and AI-2 molecule synthesis (namely *lux*S). In addition, these lactobacilli strains have been observed to support the phagocytic activity of macrophages against *C*. *jejuni* and stimulate the production of immune mediators like IFN-γ, IL-1β, IL-12p40, IL-10, and chemokine (CXCLi2) within these cells. Furthermore, *L*. *salivarius*, *L*. *reuteri*, *L*. *crispatus*, and a combined formulation of these lactobacilli exhibited potential in amplifying the macrophage expression of co-stimulatory markers, including CD40, CD80, and CD86.

We have extensively shown that various concentrations of antimicrobial mixtures, including 0.1% or 0.5%, are able to prevent *C*. *jejuni* and *E*. *coli* attachment and infection of epithelial cells [28,29]. We have shown in these studies that mixtures of natural antimicrobials are able to disrupt molecular mechanisms at the host or bacterial level, preventing host inflammatory events and reducing the negative impact on animal health [30]. Therefore, this study aims to investigate the effect of organic acid-based mixtures (Ac) on the ability *C*. *jejuni* and *E*. *coli* to attach and form biofilm on various surfaces, including plastic, chicken carcass skins, straw bedding, and eggshells. Moreover, on one level, we aimed to assess the effects of antimicrobials on the expression of genes involved in attachment and biofilm formation on different surfaces in *E*. *coli* (l*uxS*, *fimC*, *csgD*) and *C*. *jejuni* (*luxS*, *flaA*, *flaB*) and, on a second level, we aimed to observe if any of the polysaccharidic surface structures are affected. 

## 2. Materials and Methods

### 2.1. Bacterial Strains and Growth Medium and Antimicrobial Mixture

*Escherichia coli* NCTC K12 and *Campylobacter jejuni* NCTC 11168 were used to assess biofilm formation in 6-well plates. Trypticase Soy Broth with 0.6% Yeast Extract medium (TSBYE) and Mueller Hinton Broth (MHB) supplemented with 10% foetal bovine serum (FBS, Sigma-Aldrich, Gillingham, UK) were used to grow the *E*. *coli* NCTC 10538 K12 and *C*. *jejuni* NCTC 11168 strains, respectively. *C*. *jejuni* and *E*. *coli* growth curves were performed as previously described [31]. Briefly, at regular time intervals, 1 mL samples were removed and immediately diluted and plated onto MH agar for counting *Campylobacter* and onto TSBYE for *E*. *coli*. The antimicrobial mixture, Auracount (Ac), contained 5% maltodextrin, 1% sodium chloride, 42% citric acid, 18% sodium citrate, 10% silica, 12% malic acid, 9% citrus extract, and 3% olive extract (*w*/*w*). The raw materials were supplied by Bioscience Nutrition Ireland, Limerick, Ireland.

### 2.2. Biofilm Formation

#### 2.2.1. *E. coli* and *C. jejuni* Biofilm Formation in 6-Well Plates

*E. coli* K12 agar-grown colonies were used to inoculate 10 mL of TSBYE medium, and the strain was incubated overnight at 37 °C. After 24 h, the overnight culture was diluted 1:100, washed twice with TSBYE, and centrifuged for 10 min at 7000 rpm. To determine the impact of the natural antimicrobial mixtures (Ac) on *E. coli* biofilm formation at concentrations of 0.5% and 0.1%. Media, including antimicrobials, were added to each 6-well plate in a volume of 2 mL/well. To avoid evaporation of the inoculum from the wells, each plate was covered with an adhesive seal (Thermo Scientific, Waltham, MA, USA) and further incubated at 37 °C for 24 h. Colonies of *C. jejuni* 11168 were grown on Blood Agar Base Nr. 2 (BAB, OXoid Ltd., Hampshire, UK) with the inclusion of 5% (*v*/*v*) defibrinated horse blood (Aquilant Scientific, Newtownards, UK) and incubated under microaerophilic conditions at 42 °C for 48h. Colonies were gently harvested and diluted to 0.4 optical density (OD) in MHB containing 10% FBS. Bacteria were washed twice with MHB and centrifuged for 10 min at 7000 rpm. Concentrations of 0.5% and 1% Ac were added to MHB supplemented with 10% FBS. Bacterial medium with treatments was added to each *C. jejuni* pellet and inoculated in a 6-well plate (2 mL/well), covered with an adhesive seal, and incubated at 42 °C for another 72 h. 

#### 2.2.2. *E. coli* and *C. jejuni* Biofilm Formation on Chicken Carcasses

The procedure is graphically described in Figure 4A,B. Following inoculation and Ac treatment, the chicken carcasses were gently washed to remove un-attached bacteria, and each carcass was introduced in a stomacher bag to remove skin-attached bacteria. The emulsified, skin-attached bacteria was collected by centrifugation at 4000 rpm. The BS EN ISO10272-1:2006 was followed as previously described [32]. The resulting pellet was plated on modified charcoal cefoperazone deoxycholate agar (mCCDA) and incubated at 41.5 °C until single colonies were countable. To confirm that the resulting colonies represent a typical Campylobacter colony, the motility and oxidase tests were performed. DNA was extracted from each individual isolate using half of a 10 μL loopful in 1 mL of SET buffer (150 mmol/L^−1^ NaCl, 15 mmol/L^−1^ EDTA, 10 mmol/L^−1^ Tris–HCl, pH 8.0). A similar protocol was followed for *E. coli* enumeration and performed as previously described [33].

#### 2.2.3. *E. coli* and *C. jejuni* Biofilm Formation on Straw Bedding and Eggshells Sprayed with Ac

Straw bedding (200 g) was used to investigate the impact of Ac spraying on *E. coli* and *C. jejuni* attachment. The experiment was performed in triplicates and detailed in Figure 4A,B. Straw bedding was exposed to UV light for 5 min to kill the background microbial flora before the application. Bacterial strains were grown as described above and diluted to concentration of 4 × 10^8^ CFU/mL. Straws, sprayed with 30 mL of 0.1%, 0.5% Ac, or un-sprayed, were inoculated with 1 mL of the bacterial suspension and incubated for 24 h in the case of *E. coli* or 72 h in the case of *C. jejuni*. Spraying was performed with a manual spray gun equipped with a 0.5 mm nozzle. After incubation, straws were then inserted in a 0.5 L stomacher bag containing 500 mL PBS buffer and stomached for 5 min to release the attached bacteria. The PBS buffer containing bacteria was collected, and bacteria were harvested by centrifugation for 30 min at 4000 rpm. Un-treated straws were used as positive controls. Harvested bacteria were further used to quantify gene expression and enumeration. The impact of Ac on eggshell attachment was performed as described in Figure 4 and was investigated in two ways. Firstly, we have investigated the ability of Ac to prevent attachment to eggshells in a fluid environment containing either 0.1% or 0.5% Ac, as described in Figure 4A,B. Secondly, eggshells initially cleaned with 70% ethanol and washed with sterile dH_2_O were sprayed with a solution of 0.1% or 0.5% Ac and incubated for 30 min. After the 30 min incubation, eggs were sprayed with either 10^3^ *C. jejuni* or 10^3^ *E. coli*. Inoculated eggshells were incubated for 72 h in the case of *C. jejuni* or 24 h for *E. coli*. Next, eggshell-attached bacteria were removed by washing with sterile PBS buffer and further used to investigate gene expression and potential bacterial growth. To ensure that sufficient bacterial pellet is generated for gene expression, bacteria from five separate experiments were isolated, and a pooled sample was generated. Samples were analysed individually to quantify total attachment.

### 2.3. Crystal Violet Assay

After biofilm formation in the presence of Ac, the supernatant was removed by gently washing the plates twice with 2 mL of phosphate-buffered saline (PBS) and then air-dried. Subsequently, 2 mL of methanol was added to each well to fix the adherent bacteria. After 2 min, the methanol was removed, and the plates were washed twice with sterile PBS and air-dried. The next step was crystal violet (CV) staining, in which the volume of 2.25 mL of 0.1% CV solution was added to all wells. After 10 min of dyeing, CV was removed, and the wells were washed twice with PBS and dried. In the last step, 2 mL of 30% glacial acetic acid was added to each well and incubated for 10 min. The content of each plate was moved carefully, without agitation, into a new plate, and the absorbance was measured using a microplate reader (FluoStar Omega, Premier Scientific, Belfast, UK) at an absorbance of 550 nm, and 30% glacial acetic acid was used as a blank. All the steps were performed at room temperature. The assay was repeated thrice on 3 three separate occasions. 

### 2.4. Total RNA Isolation

The plates containing the bacteria were removed from the incubator after expiration. The supernatants from each treatment were used for optical density measurements using a Jenway 7315 spectrophotometre (Bibby Scientific Ltd., Stone, UK) at 600 nm to obtain an 0.4 OD. For biofilm formation, the remaining supernatants were removed from the plates, and each well was gently washed twice with 2 mL of PBS. To remove the biofilm from the wells, the addition of 500 µL PBS was added to each well and harvested with a cell scraper. The resulting solutions were inserted into a spectrophotometre and subjected to optical density measurement. Afterward, each ml of supernatant/biofilm solution was centrifuged for 2 min at 10,000 rpm to facilitate the pellet formation at the bottom of the centrifuge tube. To lyse the cells, 350 µL of Buffer RLT Plus (RNeasyPlus Mini Kit; Qiagen, Manchester, UK) was added to each pellet, vortexed for 30 s, and sonicated in an ultrasonic processor for 2–3 s at an amplitude of 70%. The metal rod of the sonicator was washed with RNazeZAP™ (Thermo Fisher, Horsham, UK) to avoid contamination of the samples. Total RNA was isolated following the manufacturer’s protocol using an RNeasyPlus Mini Kit (Qiagen, Manchester, UK). The purity of the RNA isolates was measured using a NanoDrop 1000 UV/VIS Spectrophotometre (Thermo Fisher, Horsham, UK). 

### 2.5. cDNA Conversion

Complementary DNA (cDNA) was obtained by a standard RT-PCR Procedure using single reactions with random hexamer primers (Roche, East Sussex, UK). For this protocol, we used the components listed in Table 1 (Part A), which were placed on ice. All reagents were briefly centrifuged before setting up the reaction and then added to a nuclease-free microcentrifuge tube. After mixing and centrifugation, the sample was subjected to denaturation of the template–primer mixture by heating the tubes for 10 min at +65 °C in a block cycler (Prime Thermal Cycler, Bibby Scientific Ltd., Stone, UK) with a heated lid. Then, the tubes were immediately cooled on ice blocks, and the components of the RT mix were added to the tube containing the template–primer mix (Table 1, Part B).

All reagents were briefly mixed for a few seconds to collect the samples at the bottom of the tubes (SIGMA 2-16 KL centrifuge, Sigma-Aldrich, Gillingham, UK). Subsequently, all tubes were placed in a thermal block cycler with a heated lid and incubated the RT reaction for 10 min at 25 °C, followed by 30 min at 55 °C (Prime Thermal Cycler; Bibby Scientific, Ltd., Stone, UK). To inactivate Transcriptor Reverse Transcriptase, the lid was heated to 85 °C for 5 min, immediately chilled on ice, and stored in a freezer at −20 °C. The assays were performed in triplicate. Finally, the purity of the RNA isolates was measured using a NanoDrop spectrophotometre (NanoDrop 1000™ UV/VIS Spectrophotometre, Thermo Fisher, USA).

### 2.6. Gene Expression Analysis (SYBR^®^Green Assays)

Quantitative real-time PCR (qRT-PCR) was performed to assess the relative expression levels of target genes. Primers used in this study are listed in Table 2 and Table 3. The relative quantity of mRNA was determined by the double-delta CT (DDCT) method. The rrsA 16S rRNA and 16S rRNA genes were used as the housekeeping genes in *E. coli* and *C. jejuni*, respectively. First, the difference between experimental values (experimental gene tested—housekeeping experimental gene) and control values (test gene control—housekeeping gene control) was calculated, which were the Δ^Ct^ values for the experimental (∆^CTE^) and control (∆^CTC^) conditions, respectively. The double delta CT (ΔΔCT) value was calculated as the difference between the experimental values and control conditions. Thereafter, the 2^−ΔΔCt^ value was calculated to determine the fold change in gene expression.

To identify the target genes by qRT-PCR (CFX96 Deep well™ Real-Time PCR Detection System, Bio-Rad, Singapore), SYBR^®^Green dye (Qiagen, UK) was used. To 10 µL of SYBR Green master mixture were added 5.2 µL of sterile water (PCR grade), sequence-specific primers in the volume of 1.4 µL (10 µM), and 2 µL of the DNA template in a final reaction volume of 20 µL. The PCR conditions are presented in Table 4.

### 2.7. Bacterial Surface Polysaccharide Preparation

CPS was prepared from bacteria by using a method described by [38]. Briefly, bacteria were harvested, centrifuged, and resuspended in 100 μL of 31.25 mM Tris-HCl (pH 6.8), 4% sodium dodecyl heated at 100 °C for 5 min, and 1 μL of 20 mg ml−1 proteinase K was added to the solution, and the tubes were incubated for 1 h at 50 °C. The samples were fractionated on NuPage Novex 10% bis-Tris gels (Thermo Fisher, Horsham, UK). Following electrophoresis, gels were stained with Alcian blue (0.1% Alcian blue in 40% ethanol, 5% acetic acid).

### 2.8. Statistical Analysis

Statistical analyses were performed using GraphPad Prism 9 software. Data were represented as mean  ±  SD. *p*-values  <  0.05 were considered statistically significant following estimations using the Student’s *t* was used.

## 3. Results

### 3.1. In Vitro Bacterial Growth Profiles in the Presence of 0.1% and 0.5% Ac

**Figure 1:**
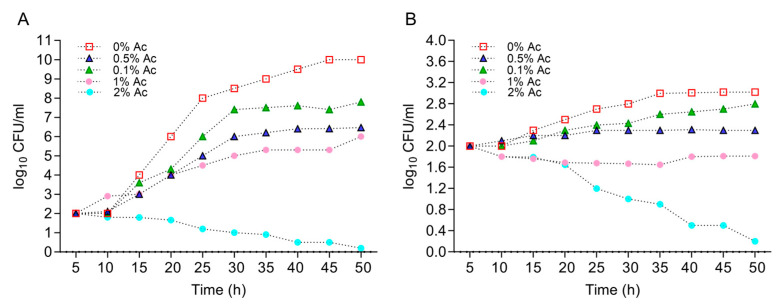
The impact of Ac (0.1%, 0.5%, 1%, and 2%) on the growth of *E. coli* K12 (**A**) and *C. jejuni* 11168 (**B**). Triplicate experiments were performed. In order to quantify the growth, the absorbance was measured at 600 nm every 0.5 h for 24 h.

First, we have characterized the growth profiles of *E. coli* K12 and *C. jejuni* 11168 at various concentrations of Ac (0.1%, 0.5%, 1%, and 2%) in order to identify the concentration that will reduce bacterial growth but with no lethal impact. This information was necessary to identify the changes in bacterial biofilm formation and surface structure changes in the presence of Ac. Figure 1A shows that Ac disrupted the growth of *E. coli* K12 at concentrations of 0.1%, 0.5%, and 1%, but with lethal effects at 2%. Similar effects were observed for *C. jejuni* 11168 with the difference that at 1% Ac, the growth was flat, and lethal effects were detected at 2% (Figure 1B). Based on these results, we have decided to further investigate the impact of 0.1% and 0.5% Ac on *E. coli* K12’s ability to attach to non-organic and organic surfaces, the expression of genes involved in bacterial attachment, and the profiles of bacterial surface structures with impact on attachment.

### 3.2. In Vitro Bacterial Biofilm Formation in the Presence of 0.1% and 0.5% Ac

**Figure 2:**
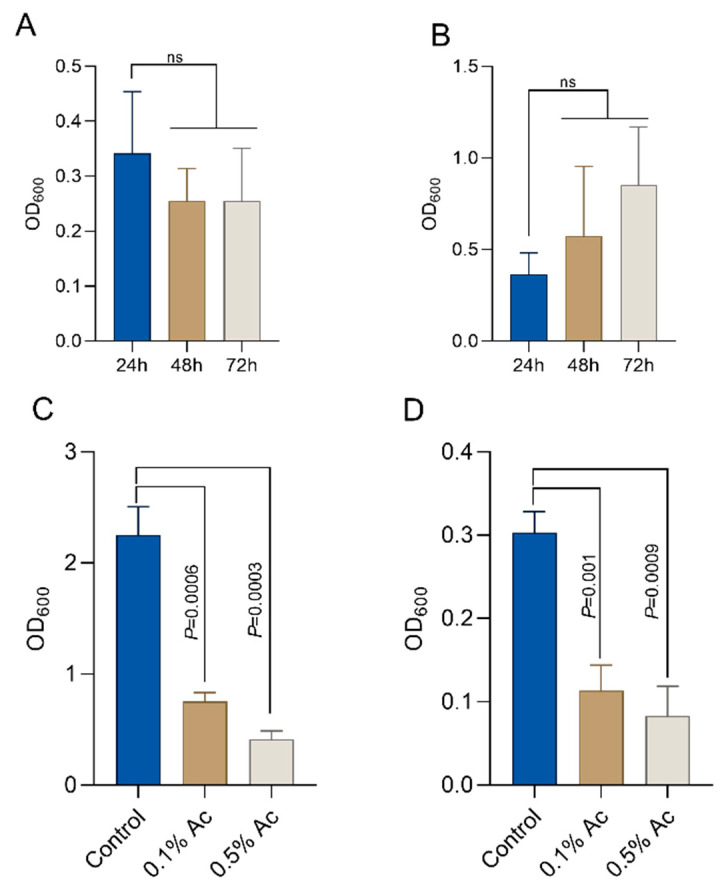
Biofilm formation by *E. coli* K12 ((**A**)—24h) and *Campylobacter jejuni* 11168 ((**B**)—72h). In the presence of 0.5% and 1%, Ac was able to significantly reduce the ability of *E. coli* K12 (**C**) and *C. jejuni* 11168 (**D**) to form biofilm. Significant differences were analysed using Student’s *t*, and error bars represent the standard deviation of means from three different experiments, each containing triplicate samples. *P* values are indicated on graphs (ns—not significant).

To quantify the impact of Ac on *E*. *coli* and *C*. *jejuni* biofilm formation, we aimed to establish the baseline biofilm formation for both bacteria in the absence of the natural antimicrobial. As indicated in Figure 2, *E*. *coli* established a significant biofilm after 24 h of growth (Figure 2A) and *C*. *jejuni* after 72 h of growth (Figure 2B). Next, we investigated the impact of the natural antimicrobial mixture (Ac) on the ability of *E*. *coli* and *C*. *jejuni* to form biofilm. Concentrations of 0.1% and 0.5% Ac were present in the culture medium during bacterial biofilm formation. Our results show that Ac can reduce *E*. *coli* (Figure 2C) and *C*. *jejuni* (Figure 2D) attachment to the surface of 6-well plates in which the bacterial biofilms were grown. These results indicate that subinhibitory concentrations of natural antimicrobial mixtures can prevent bacterial surface attachment, potentially by inhibiting molecular mechanisms involved in biofilm formation, such as EPS production, which plays an important role in bacterial integrity and survival.

### 3.3. The Impact of Ac on Bacterial Gene Expression in Biofilm and Planktonic Cells

**Figure 3:**
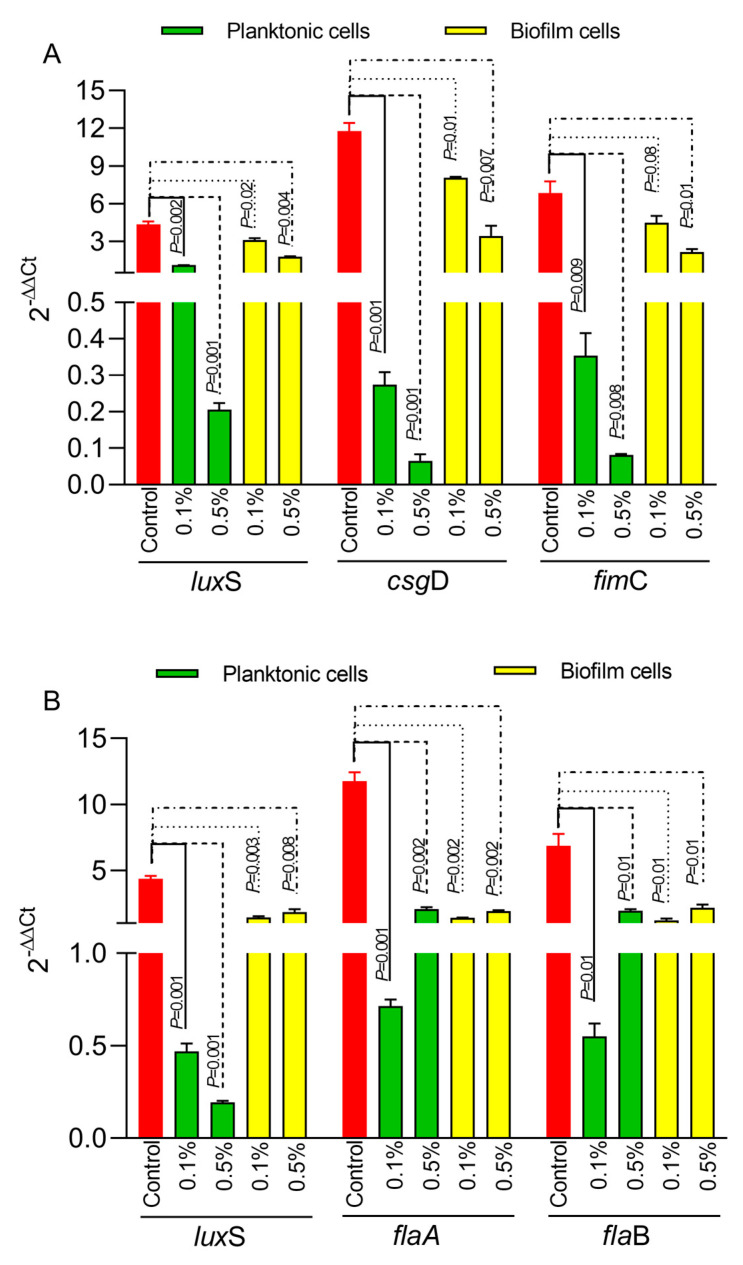
The impact of 0.5% or 1% Ac on *E coli* (**A**) and *C. jejuni* (**B**) *lux*S, *fim*C, *csg*D, *fla*A, *fla*B gene expression in biofilm and planktonic cells. Data are expressed as 2^−DDCt^ values, which are the mean of three test replicates. Student’s *t*-test was used to statistically compare the effect of Ac. *p* values are indicated on the graphs.

Next, we have investigated the impact of Ac on bacterial biofilm gene expression in both *E*. *coli* and *C*. *jejuni*. The expression of the *lux*S quorum-sensing gene in *E*. *coli* (Figure 3A) was significantly downregulated in both planktonic cells and in the biofilm cells at both concentrations (0.5% and 0.1%). Similar results were observed for the gene responsible for Type 1 fimbriae, *fim*C (Figure 3A), and for the *csg*D gene, which is responsible for the production of curli fibers and cellulose essential in the extracellular matrix of the *E*. *coli* bacterial biofilm. In the case of *C*. *jejuni*, the expression of the *lux*S gene (Figure 3B) was also significantly downregulated in both planktonic and biofilm cells. The expression of the *fla*A and *fla*B genes (Figure 3B) was downregulated at both concentrations in planktonic and biofilm cells. 

### 3.4. The Role of Ac in Mediating C. jejuni 11168 and E. coli K12 Attachment Chicken Skins

**Figure 4:**
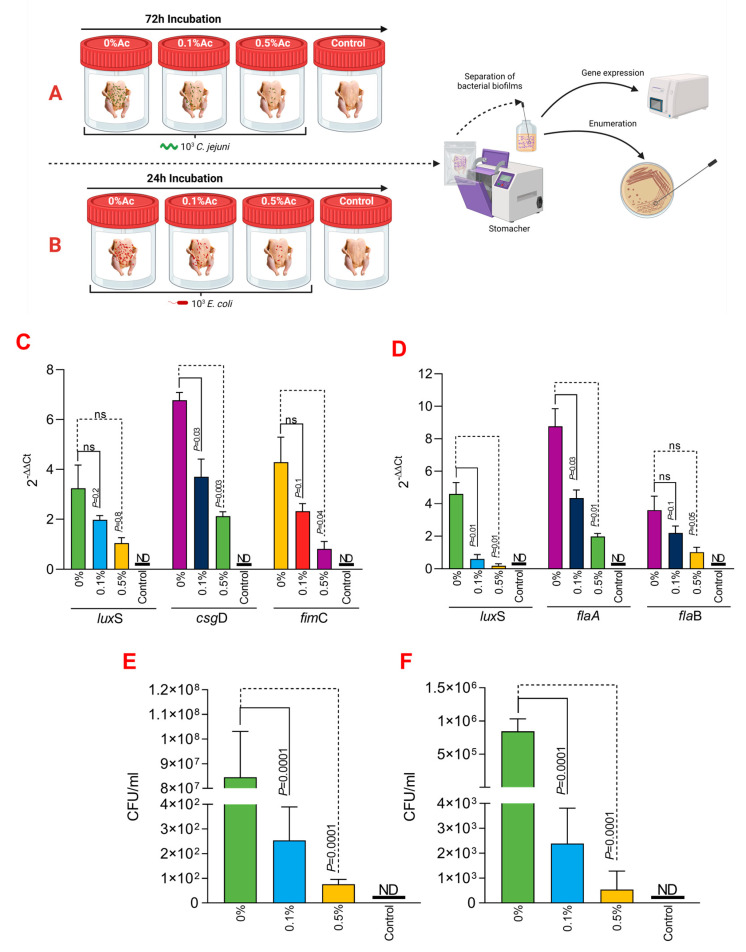
In vitro investigation of 0.1% and 0.5% Ac impact on the ability of *C. jejuni* 11168 and *E. coli* K12 *lux*S, *fim*C, *csg*D, *fla*A, *fla*B gene expression and attachment to chicken carcass skins. (**A**) describes the experimental design for *C. jejuni* 11168, and (**B**) shows the experimental design for *E. coli* K12. (**C**) represents the gene expression profiles of *E. coli* K12 and (**D**) for *C. jejuni* 11168 attached to chicken carcass skins. Total bacterial attachment to chicken skins is represented in (**E**) for *E. coli* K12 and in (**F**) for *C. jejuni*. Data originate from five individual experiments, and the significance values are indicated on graphs. (**A**,**B**) were designed using Biorender.com. ND—not detected, ns—not significant.

To further expand on our observations, we have next investigated its ability to reduce *C*. *jejuni* 11168 and *E*. *coli* K12’s ability to attach to chicken skins. As presented in Figure 4A,B, we have designed an in vitro experiment which has allowed us to incubate the chicken skins in the presence of 0.1% or 0.5% Ac, followed by isolation of the attached bacteria and investigation of *lux*S, *fim*C, *csg*D, *fla*A, *fla*B gene expression and quantification of total bacterial adhesion. The expression of the *lux*S quorum-sensing gene in *E*. *coli* (Figure 4C) was significantly downregulated at both Ac concentrations (0.5% and 0.1% Ac). The results were similar for *fim*C; however, even though a decreasing trend was also observed for the *csg*D gene, the differences were only significant at 0.5% Ac. For *C*. *jejuni*, the expression of *lux*S and *fla*A gene (Figure 4D) was also significantly downregulated at both concentrations. As for *fla*B, the decreasing trend in gene expression was also observed but at no statistical significance. The decrease observed in the *lux*S, *fim*C, *csg*D, *fla*A, and *fla*B gene expression was associated with a significant decrease in bacterial attachment to chicken skins for both *C*. *jejuni* 11168 (Figure 4E) and *E*. *coli* (Figure 4F). Taken together, these results suggest that natural antimicrobial mixtures, such as Ac, have the potential to be used as pathogen repellents in poultry production settings.

### 3.5. The Role of Ac in Mediating C. jejuni 11168 and E. coli K12 to Straw

**Figure 5:**
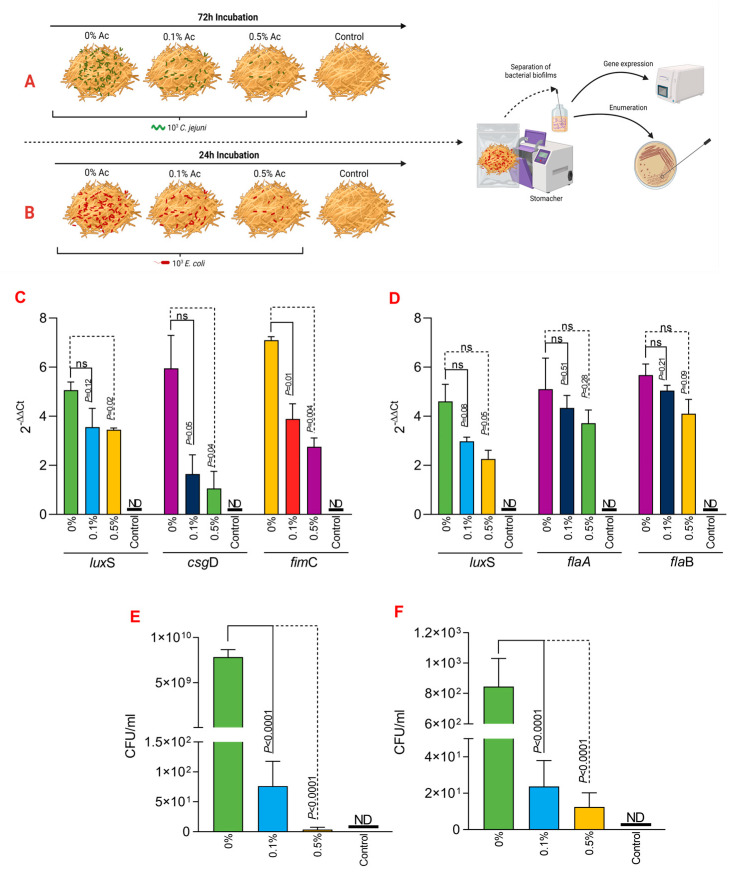
In vitro investigation of 0.1% and 0.5% Ac impact on the ability of *C. jejuni* 11168 (**A**) and *E. coli* K12 (**B**) *lux*S, *fim*C, *csg*D, *fla*A, *fla*B gene expression and attachment to straw. Gene expression values of *E. coli* K12 (**C**) and *C. jejuni* 11168 (**D**) attached to straw are indicated. Total bacterial attachment to straw is represented in (**E**) for *E. coli* K12 and in (**F**) for *C. jejuni*. Data originate from five individual experiments, and the significance values are indicated on graphs. (**A**,**B**) were designed using Biorender.com. ND—not detected, ns—not significant.

Next, we investigated if Ac can also reduce the attachment of *C*. *jejuni* 11168 and *E coli* K12 to irradiated straw. Figure 5A,B represent a graphical description of the experimental design used to investigate the impact of 0.1% or 0.5% Ac on the bacterial attachment to the straw surface. Following the quantification of straw-attached bacteria, we have further investigated the *lux*S, *fim*C, *csg*D, *fla*A, and flaB bacterial gene expression. Our data shows that the expression of the *E*. *coli* K12 *lux*S gene was not significant when 0.1% Ac was used; however, it was significantly downregulated at 0.5% (Figure 5C). The results were similar for *fim*C where significance was observed at both Ac concentrations, unlike *csg*D, where the results were only significant when the straw was sprayed with 0.5% Ac (Figure 5C). Total *E*. *coli* attachment, following Ac spraying of straw, was also significantly reduced (Figure 5E). Nonetheless, the impact of Ac on the expression of *lux*S, *fla*A, and *fla*B in *C*. *jejuni* had no statistical significance (Figure 5D); however, the overall attachment to straw was significantly reduced, suggesting that other bacterial surface structures involved in the attachment might be affected.

### 3.6. The Role of Ac in Mediating C. jejuni 11168 and E. coli K12 to Eggshells

**Figure 6:**
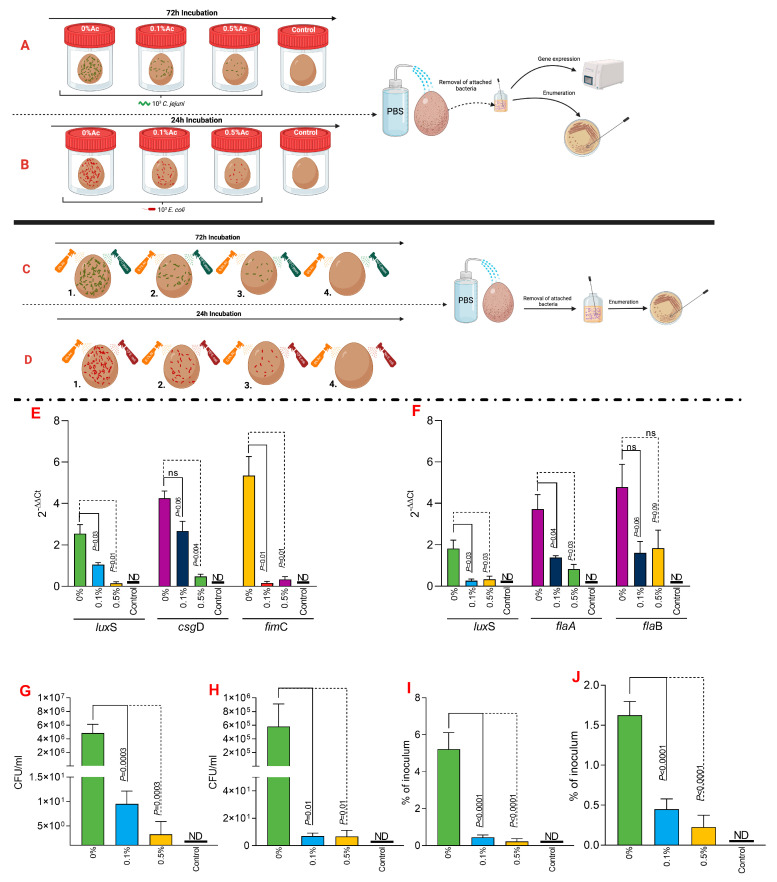
In vitro investigation of 0.1% and 0.5% Ac impact on the ability of *C. jejuni* 11168 (**A**) and *E. coli* K12 (**B**) *lux*S, *fim*C, *csg*D, *fla*A, *fla*B gene expression and attachment to eggshell surface during liquid incubation. A similar experiment was designed to study their attachement during straying, *C. jejuni* 11168 (**C**) and *E. coli* K12 (**D**). *E. coli* K12 (**E**) and *C. jejuni* 11168 (**F**) gene expression indicated. Total bacterial attachment to eggshells is indicated in (**E**) for *E. coli* K12 and in panel (**F**) for *C. jejuni*. The total bacterial attachment during fluid incubation for both *E. coli* (**G**) and *C. jejuni* (**H**). Panel (**I**) represent the percentage of the initial inoculum attached to eggshell surface pre-sprayed with 0.1% or 0.5% Ac for *E. coli* K12 and (**J**) for *C. jejuni* 11168. Data originate from five individual experiments, and the significance values are indicated on graphs. (**A**–**D**) were designed using Biorender.com. ND—not detected, ns—not significant.

To investigate the impact of Ac on bacterial attachment to eggshells, we have taken two separate approaches, as graphically presented in Figure 6A,B (fluid incubation) and in Figure 6C,D (sprayed eggshell). The fluid experimental design aimed to explore if *C*. *jejuni* 11168 or *E*. *coli* K12 grown in a liquid environment and in the presence of 0.1% or 0.5% Ac will exhibit a reduced ability to attach to the eggshell surface. Our results show that the expression of *lux*S, *csg*D, or *fim*C genes involved in the eggshell attachment of *E*. *coli* was significantly downregulated, except for *csg*D, where significance was not detected at 0.1% Ac (Figure 6E). Gene expression results were similar for *C*. *jejuni* 11168, except for *fla*B, where significant downregulation was not detected (Figure 6F). The total bacterial attachment during fluid incubation (Figure 6A,B) was significantly downregulated for both *E*. *coli* (Figure 6G) and *C*. *jejuni* (Figure 6H). These results clearly indicate that Ac can prevent pathogen attachment. Our next approach aimed to elucidate if pre-praying of eggshells, followed by bacterial sprayed inoculation, will reduce the persistence of bacteria, measured as a percentage of the initial inoculum. The results presented in Figure 6 clearly show that a significantly reduced amount of *E*. *coli* (Figure 6I) or *C*. *jejuni* (Figure 6J) initial inoculum was able to survive on the surface of the eggshell. Overall, the data presented here clearly suggest that natural antimicrobials have the potential to be used to prevent bacterial attachment and survival on the shell surface.

### 3.7. Changes in C. jejuni and E. coli Surface Polysaccharides in the Presence of 0.1% Ac and 0.5 Ac during Attachment to Chicken Skins, Straws, and Eggshells

**Figure 7:**
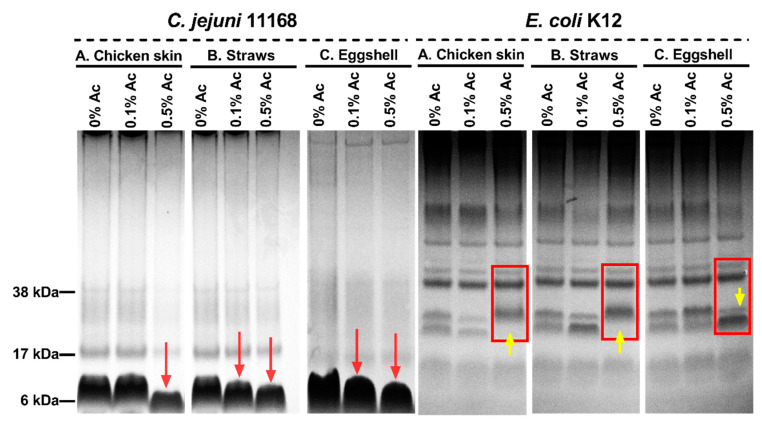
Alcian blue-stained surface polysaccharides of *C. jejuni* 11168 and *E. coli* K12 in 10% bis-Tris gels following exposure 0% Ac, 0.1% Ac, or 0.5% Ac and attached to chicken skin (**A**), straws (**B**), and eggshells (**C**). Red arrows indicate changes in the molecular weights of *C. jejuni* surface polysaccharides, and yellow arrows indicate changes in *E. coli* surface polysaccharidic structures.

We then explored the hypothesis that structural changes in bacterial surface polysaccharides might be responsible for reduced bacterial attachment in the presence of 0.1% and 0.5% Ac. As shown in Figure 7, the surface polysaccharides of Ac-exposed bacteria suffered structural changes as indicated by Alcian blue extracts separated by electrophoresis. In the case of *C. jejuni* isolated from chicken skin, structural changes were only detected at 0.5% Ac, compared to bacteria isolated from straws and eggshells, where structural changes were observed at both 0.1% and 0.5% Ac (as indicated by red arrows). The surface polysaccharides of *E. coli* K12 were also affected by the presence of Ac. As indicated in Figure 7, at 0.5% Ac (yellow arrow), significant changes are observed in bacteria attached to chicken skin and straw, where the absence of stained bands is noticed. Contrary to the chicken skin and straw attached *E. coli* K12, the changes in surface polysaccharides of bacteria attached to eggshells are characterized by an increased presence of stained bands (polysaccharide expression) at 0.5% Ac. The observed changes in bacterial surface polysaccharides strongly suggest that Ac can structurally modify the bacterial surface structures and impact their ability to attach and potentially form biofilm.

## 4. Discussion

According to the World Health Organization, *Escherichia* and *Campylobacter* spp. are the most common foodborne pathogens, affecting millions of people annually. *Campylobacter* spp. is primarily responsible for foodborne illnesses caused by raw milk, raw or undercooked poultry, and contaminated drinking water, whereas *E*. *coli* is associated with undercooked meat, unprocessed milk, and contaminated fresh fruits and vegetables. Last but not least, these bacteria can develop biofilm on food processing surfaces and equipment, increasing the risk of foodborne outbreaks [39,40]. As such, reducing biofilm formation by both *C*. *jejuni* and *E*. *coli* will enhance food safety, mitigate antibiotic resistance, and prevent foodborne illnesses.

Herein, we evaluated the effect of natural-based antimicrobials against two pathogenic strains of *Escherichia coli* NCTC 10538 K12 and *Campylobacter jejuni* NCTC 11168 and assessed their capacity to impede surface attachment. We showed that natural antimicrobials could reduce their adherence to the surface, modify the expression (*E*. *coli*—*lux*S, *fim*C, *csg*D, and *C*. *jejuni*—*lux*S, *fla*A, *fla*B) of genes involved in biofilm formation, and potentially modify the bacterial surface structures involved in attachment. Moreover, our data show that gene expression downregulation is associated with a reduced ability to attach to organic and non-organic surfaces in a dose-dependent manner. L*uxS* gene, involved in quorum sensing and coding for a protein crucial for *C*. *jejuni* adaptation to environmental conditions [41], expression of virulence factors [42], and biofilm formation [43], is considered a promising target for controlling *C*. *jejuni* infections. Natural antimicrobials inhibit *C*. *jejuni* by blocking efflux pump activity and quorum sensing and impact host colonization. Citrus extracts, such as *Citrus limon*, *Citrus aurantium*, and *Citrus medica*, have been found to inhibit the quorum-sensing molecule AI-2 by 90%, resulting in reduced motility and biofilm formation [44]. These results further demonstrate the ability of natural phenolic compounds to disrupt quorum sensing in *C*. *jejuni*, thereby reducing its fitness. Similarly, antimicrobials can activate the AI-2 system of *E*. *coli*, promoting biofilm formation and enhancing motility [16]. Sublethal concentrations of natural antimicrobials can activate the *luxS*/AI-2 pathway, leading to higher expression of *luxS* and potentially regulating other genes responsible for biofilm formation. Furthermore, subinhibitory concentrations of natural antimicrobials, including lactic acid, citric acid, and citrus extracts, have been found to reduce *E*. *coli* O157 biofilm formation in a dose-dependent manner. Essential oils from *Thymus daenensis* and *Satureja hortensis* can inhibit bacterial growth, biofilm formation, and QS by EHEC, with gene expression analysis showing a significant reduction in *luxS* levels at MIC/2 concentrations [45]. 

Other bacterial genes, encoding flagellins and flagellar biosynthesis proteins, are also essential during the early stages of *C*. *jejuni* biofilm formation, according to a study by Kim et al. [46], and also have been shown to have reduced expression when exposed to Ac in our study. The flagellin A (*flaA*) gene is a significant virulence marker that is associated with flagella, bacterial motility, adhesion, and invasion, as noted by Bhunia [47]. Phytochemicals, such as Carvacrol and eugenol, have been observed to inhibit biofilm formation in *C*. *jejuni*, rapidly inactivate mature biofilms of the pathogen, and downregulate the motility gene *flaA* [48]. Subinhibitory concentrations of trans-cinnamaldehyde, eugenol, and Carvacrol have also been shown to affect *C*. *jejuni* biofilm formation, significantly reducing its development by 0.5 and 0.7 Log CFU/mL, respectively [24]. The best results were obtained using Carvacrol, which reduced biofilm formation to 0.75 and 1.5 Log CFU/mL after 48 h. Subinhibitory levels of trans-cinnamaldehyde significantly modulated the expression of the gene encoding motility (*flaA*) and downregulated its expression 11.7 times. Carvacrol showed similar results, reducing the expression of the *flaA* gene and obtaining values of 0.8 Log. Eugenol also downregulated the expression of the *flaA* gene, but to a lesser extent, with a two-fold reduction. The use of phytochemicals, such as beta-resorcylic acid and eugenol, at sub-inhibitory concentrations of 125 µg/mL showed promising results against 345 *C*. *jejuni* isolates from humans and broiler chickens, as reported by Ammar [49]. Eugenol was found to inhibit bacterial invasion of chicken intestinal epithelial cells by 29.16–31.94%, while beta-resorcylic acid inhibited 38.19–41.66%. Additionally, the *flaA* gene expression level was significantly downregulated after exposure to sub-inhibitory concentrations of the tested phytochemicals, achieving gene suppression of up to 0.3015-fold for beta-resolcylic acid and 0.6690-fold for eugenol.

Motility is a characteristic property of *C*. *jejuni* and is essential for efficient host colonization [50]. The *flaB* gene, also affected by Ac, is an essential component of an integral defense mechanism against infection, and disruption of this gene could prevent the development of a resistant, non-motile phenotype during infection, a gene also downregulated in our study in the presence of Ac [51]. Other studies have confirmed the efficiency of antimicrobials used in mixtures. For example, citrus extracts have been shown to decrease motility and biofilm formation in *C*. *jejuni* [44] by adding 75% MBC of the three extracts. Also, these extracts were able to significantly reduce the expression of the *flaB* gene, and the best results were obtained in the case of the *C*. *limon* extract, having the highest level of suppression. Moreover, all extracts suppressed *flaB* gene expression to undetectable levels in *Campylobacter jejuni* NCTC 11168. Blackberry and blueberry pomace extracts did not significantly alter *flaB* gene expression in *C*. *jejuni* RM1221 [52]. In mixed culture conditions between *L*. *casei* and conjugated linoleic acids (LC-CLA) in the presence of berry pomace phenolic extract (BPPE) had the ability to reduce the growth of *C*: *jejuni* RM1221 by more than 3 Log CFU/mL in 48 h, and the expression of the *flaB* gene was up-regulated reaching values up to 3.4 fold [53].

Adhesive filaments promote attachment to surfaces and the formation of the extracellular matrix of bacterial biofilms [54]. Genes involved in their production (*fim*C, *csg*D) were also downregulated by Ac in our work. In a study by Kim et al., 2016 [55] 11 eugenol-related compounds were investigated. The results showed that 0.005% eugenol significantly inhibited the formation of EHEC biofilms, reaching 87%. In the same study, it has been shown that three essential oils (bay, clove, and pimento berry) had inhibitory effects on *fim*C and *csg*D but with no inhibitory effects on *E*. *coli* (BW25113, MG1655, and TG1). Analysis of the expression of genes involved in biofilm formation showed that clove oil and eugenol inhibited *csgD* expression by 7- and 8-fold, respectively. These results indicate that these two phytochemicals strongly reduced the transcription of curli genes, including fimbria type I genes. According to a study by Rathi et al. [56], 48 mM caffeine can eliminate more than 90% of the pathogenic bacteria *E*. *coli*, and the growth and development of the pathogenic biofilm are inhibited in a dose-dependent manner. The expression of the structural components of curli genes, including the *csgD* gene, was drastically reduced in the presence of caffeine, starting from the lowest concentration of 20 mM. The essential oil of *Lippia origanoides* thymol-Carvacrol II chemotype also demonstrated biological activity against *E*. *coli* ATCC 25992 [57]. This essential oil was found to inhibit biofilm formation by downregulating the expression of genes involved in cell aggregation (*csgD* gene) in *E coli*. Moreover, phenolic compounds (tannic acid, gallic acid, methyl gallate, epigallocatechin gallate) were also proven to have the ability to influence the growth-swarming motility, biofilm formation, and expression virulence genes of *E*. *coli* [58]. Tannic acid, for example, led to overexpression of the *csgD* gene; however, gallic acid caused a decrease in gene expression. Expression of the biofilm-forming gene (*csgD*) was largely unaffected by natural compounds (0.1% citral, 0.25% eugenol, and 0.5% hexanal) [59].

Natural antimicrobials can also enhance the antimicrobial effect of antibiotics. It has been shown that the tea tree essential oil nanoemulsion can enhance the activity of antibiotics against multidrug-resistant *E*. *coli* by disrupting their outer and inner membranes by inhibiting efflux pumps [60] and the mRNA expression of the *fimC* gene [61]. Moreover, the use of resveratrol at a concentration of 32 µg/mL against avian pathogenic *Escherichia coli* (APEC) inhibited biofilm formation [62]. Resveratrol acts by regulating protein levels in two-component systems, chemotaxis proteins (including the *fimC* gene). Combating multidrug-resistant extraintestinal pathogenic *Escherichia coli* cetriaxone (AXO) and cranberry pomace extracts (CRAN) appears to be an effective procedure [63]. A combination of 4 mg/mL CRAN and 4 µg/mL AXO was considered to have minimal inhibitory concentrations, and exposure of bacteria to these concentrations resulted in downregulation of virulence genes. Subinhibitory concentrations of CRAN with/without AXO caused downregulation of the gene encoding Type 1 fimbriae (*fimC*). 

## 5. Conclusions

The use of phytochemicals or their combinations represents a promising strategy to control the persistence of *E*. *coli* and *C*. *jejuni* in farming and food processing environments. Our research demonstrated that an organic acid-based mixture of antimicrobials can effectively reduce the attachment of *C*. *jejuni* 11168 and *E*. *coli* K12 to various surfaces, including organic materials (chicken skins, straws, and eggshells). Moreover, the expression of biofilm formation genes in *C*. *jejuni* and *E*. *coli* was downregulated in a dose-dependent manner. We have clear indications that the bacterial surface structures suffer modifications in the presence of Ac, suggesting that further studies are needed to clarify the mechanisms of action of natural antimicrobials.

## Figures and Tables

**Table 1:** cDNA conversion reaction mixture.

Reagent	Volume
Part A	
Total RNA	1 µL
Primer: Random Hexamer Primer, 600 pmol/µL	2 µL
Water, PCR Grade	10 µL
Total	13 µL
Part B	
Transcriptor Reverse Transcriptase Reaction Buffer, 5× conc.	4 µL
Protector RNase Inhibitor, 40 U/µL	0.5 µL
Deoxynucleotide Mix, 10 mM each	2 µL
Transriptor Reverse Transcriptase, 20 U/µL	0.5 µL
Total volume	20 µL

**Table 2:** *E. coli* primers.

Gene	Function	Primer	Reference
rrsA 16S rRNA	Housekeeping gene	F: CTCTTGCCATCGGATGTGCCCA R: CCATGTGGCTGGTCATCCTCTCA	[34]
*luxS*	QS mechanism	F: CAGATGAGCAGCGTGTTG R: GCAGTGCCAGTTCTTCGT	[16]
*fimC*	Type 1 fimbriae	F: GGTAGAAAATGCCGATGGTG R: CGTCATTTTGGGGGTAAGTGC	[35]
*csgD*	Production of fibres, and cellulose	F: CGGAATCAGCCCTCCTTACTC R: GCGCCGATACGCAGCTTAT	[36]

**Table 3:** *C. jejuni* primers.

Gene	Function	Primer	Reference
rrsA 16S rRNA	Housekeeping gene	F: AATGGCTTAACCATTAAACTGC R: AACTAAATACGTGGGTTGCG	[37]
*luxS*	Modulates QS mechanism	F: AAAATGCCAGCTCCTGCTGT R: GTGCGACAACCCATAGGTGA	Laboratory collection
*flaA*	Motility and adhesion	F: GGATGGCGATAGCAGATAGTTT R: CTCATCCATAGCCTTATCAGCA	
*flaB*	Motility and adhesion	F: ACACCAACATCGGTGCATTA R: CATCCCTGAAGCATCATCTG	

**Table 4:** PCR reaction conditions.

Gene	Function	Primer	Ramp (°/s)
Pre-incubation	1	95 °C/120 s	maximum
3-step Amp.	40	95 °C/5 s	maximum
Melt	1	60 °C/10 s	0.5

## Data Availability

The data used to support the findings of this study can be made available by the corresponding author upon request.
